# Supplementary material for: Extracellular Vesicle-Mediated Secretion of Protochlorophyllide in the Cyanobacterium Leptolyngbya boryana
Source: Plants (Basel). 2022 Mar 29;11(7):910. doi: 10.3390/plants11070910 (PMC9003413; doi:10.3390/plants11070910)
Supplement: Supplementary file 1 [file plants-11-00910-s001.zip › plants-1598340-supplementary.pdf]

Extracellular vesicle-mediated secretion of protochlorophyllide in the cyanobacterium *Leptolyngbya boryana*

Kentaro Usui [1], Haruki Yamamoto [1], Takao Oi [1], Mitsutaka Taniguchi [1], Hitoshi Mori [1] and Yuichi Fujita [1,*]

Supplementary Materials

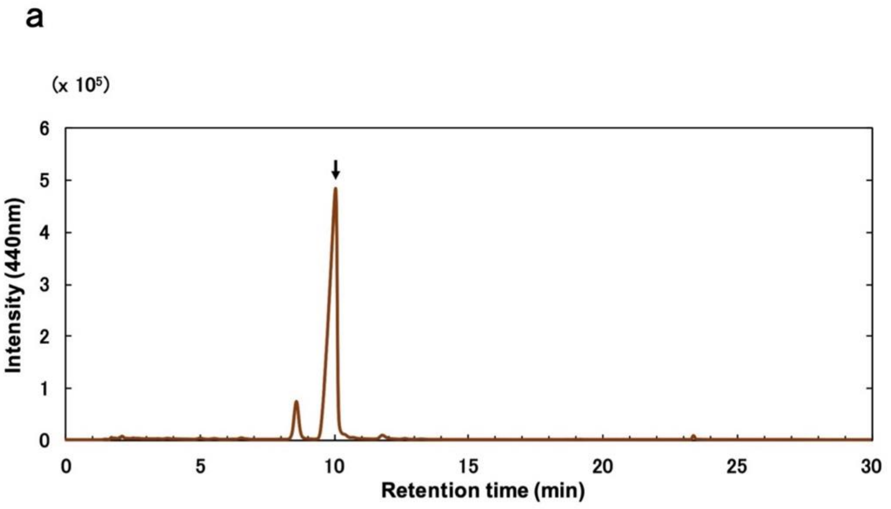

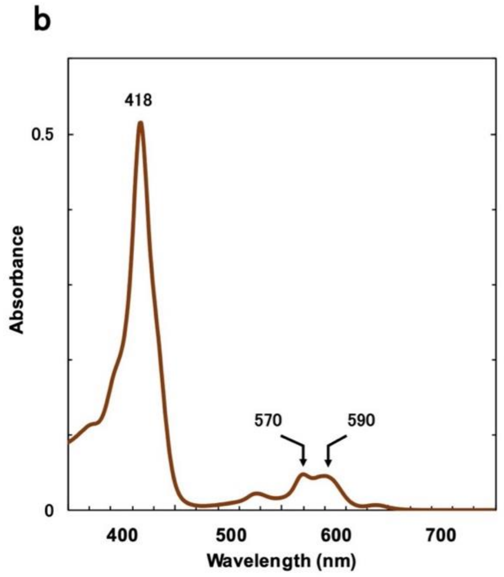

Figure S1. HPLC analysis (a) and absorption spectrum (b) of protopheophorbide that was prepared from Pchlide. Pchlide prepared from the culture medium of dark-grown YFC2 was treated by HCl (1/10 volume of 35–37% HCl was added to the Pchlide sample followed by phase-partition with ether. Protopheophorbide was eluted at 10 min (arrow, a) and the in-line absorption spectrum was shown in b.

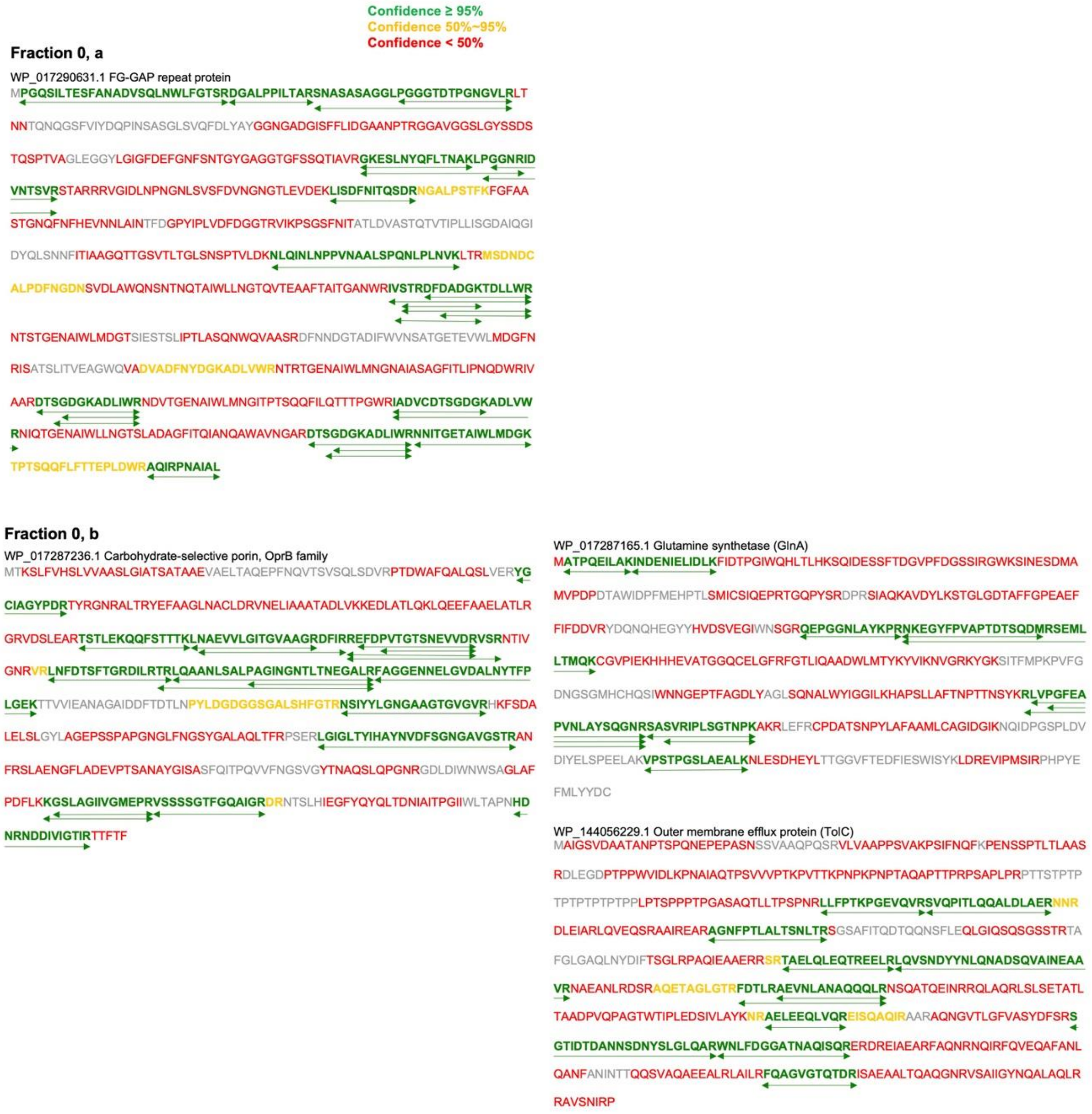
Confidence ≥ 95%
Confidence 50%–95%
Confidence < 50%
Fraction 0, a
WP_017290631.1 FG-GAP repeat protein
Fraction 0, b
WP_017287236.1 Carbohydrate-selective porin, OprB family
WP_017287165.1 Glutamine synthetase (GlnA)
WP_144056229.1 Outer membrane efflux protein (TolC)

Fraction 0, c

WP_017288757.1 Cyanobacterial porin
MSNNLWKSLLVNPVLLSATLTVSATIGIATAANASEIKADTTAPSASLDQLNAYSNEGVVSANDAGQ
VTSVSQLSDVRPTDWAFQALQSLVERYGCIAGYPDRTYRGNRALTRYEFAAGLNACLDRVNELIAA
STADLVKKEDLATLQTLQEQFAAELATLRGRVDSLEARTTTLEKQQFSTTTKLQGEAIFSISGAQGD
SKAVSSLLERRAAETGAAVARPDVDDNTTFSNRVRLSLRTSFTGQDTLLTRIQARNITDFAGAAGT
NMADLSYEGGRTADNSAFVDKLFYRFPIGQGGAVTIDAINGEFYNNVNNFNPLLASDSQGSISRFG
RFNPIYRQGASGSGSGAGVTLNYPLSQAFTVSLGYLADNGNDPTDKNGLFNGSNAALAQLAFKPS
SNIDLGFTYVRSYDRLTGASVSSGTGSALSNNPFGNFINAGGVRGSAVSADHFGVQGSFKVTPGL
VLSGWGGLTKAHAENRATNADATILNWAVGLAFPDLGGKGNMAGLIFGMPPKVTDSDLVVNGIRQ
EDEDTSFHLEGLYRLRLNDKISITPGIIVLFNPEHNNANDTVYVGTIRTTFSF

WP_017287236.1 Carbohydrate-selective porin
MTKSLFVHSLVVAASLGIATSATAAEVAELTAQEPFNQVTSVSQLSDVRPTDWAFQALQSLVERYG
CIAGYPDRTYRGNRALTRYEFAAGLNACLDRVNELIAAATADLVKKEDLATLQKLQEEFAAELATLR
GRVDSLEARTSTLEKQQFSTTTKLNAEVVLGITGVAAGRDFIRREFDPVTGTSNEVVDRVSRNTIV
GNRVRLNFDTSFTGRDILRTRLQAANLSALPAGINGNTLTNEGALRFAGGENNELGVDALNYTFP
LGEKTTVVIEANAGAIDDFTDTLNPYLDGDGGSGALSHFGTRNSIYYLGNGAAGTGVGVRHKFSD
ALELSLGYLAGEPSSPAPGNGLFNGSYGALAQLTFRPSERLGIGLTYIHAYNVDFSGNGAVGSTRA
NFRSLAENGFLADEVPTSANAYGISASFQITPQVVFNGSVGYTNAQSLQPGNRGDLDIWNWSAGLA
FPDFLKKGSLAGIIVGMEPRVSSSSGTFGQAIGRDRNTSLHIEGFYQYQLTDNIAITPGIIWLTAPNHD
NRNDDIVIGTIRTTFTF

WP_017287123.1 Carbohydrate-selective porin, OprB family
MRKILLNSLFTSPAVLGAALVMSSSAFAAQKTEVAGLPALEVGTQTTQTASVTPLTEVKSLSTEKTA
EAVKPQLQASAEFSPAKSVVVAEPVKVAQAAPEAQATPSVDSLEQINRYSREGRTANSAGQVTSV
SQLSDVRPTDWAFQALQSLVERYGCIAGYPDRTYRGNRALTRYEFAAGLNACLDRVNELIAAATAD
LVKKEDLATLQKLQEEFAAELATLRGRVDSLEARTSTLEKQQFSTTTKLAGEVVFALTDEFGVDGT
NGNNTVFQNRVRLALNTSFTGKDLLVTRIAAGNAGLFNLPAGSGAEGIQTFNFGNTGGNSGLIDWV
AYYFPVGENLKFYIPAVSGLHYDYAPTVSPALDAADGGTGPLSIFAQRNPIYLIGGGAGIGANYNLN
NRFQVSLGYLADNSTGTQLTFGANNPSDKNGLFNGSYSALGQLTFSPSEALQIGLTYVNAYRRGAI
FDGGSAVASSGTALANRNTLGTGSSQVNAYGASVAFKLSPQVVINGFFSYINANFVDDGQGKKDI
WTYGLGVAFPDFGKKGNLLGFVAGAEPYMGNPSGGASNDVPLHFEGFYKYQLTDNISITPGVIWVV
NPGQNDANDDVVIGTLRTTFTF

Fraction 0, d

WP_017287123.1 Carbohydrate-selective porin, OprB family
MRKILLNSLFTSPAVLGAALVMSSSAFAAQKTEVAGLPALEVGTQTTQTASVTPLTEVKSLSTEKTA
EAVKPQLQASAEFSPAKSVVVAEPVKVAQAAPEAQATPSVDSLEQINRYSREGRTANSAGQVTSV
SQLSDVRPTDWAFQALQSLVERYGCIAGYPDRTYRGNRALTRYEFAAGLNACLDRVNELIAAATAD
LVKKEDLATLQKLQEEFAAELATLRGRVDSLEARTSTLEKQQFSTTTKLAGEVVFALTDEFGVDGT
NGNNTVFQNRVRLALNTSFTGKDLLVTRIAAGNAGLFNLPAGSGAEGIQTFNFGNTGGNSGLIDWV
AYYFPVGENLKFYIPAVSGLHYDYAPTVSPALDAADGGTGPLSIFAQRNPIYLIGGGAGIGANYNLN
NRFQVSLGYLADNSTGTQLTFGANNPSDKNGLFNGSYSALGQLTFSPSEALQIGLTYVNAYRRGAI
FDGGSAVASSGTALANRNTLGTGSSQVNAYGASVAFKLSPQVVINGFFSYINANFVDDGQGKKDI
WTYGLGVAFPDFGKKGNLLGFVAGAEPYMGNPSGGASNDVPLHFEGFYKYQLTDNISITPGVIWV
VNPGQNDANDDVVIGTLRTTFTF

WP_017288757.1 Cyanobacterial porin
MSNNLWKSLLVNPVLLSATLTVSATIGIATAANASEIKADTTAPSASLDQLNAYSNEGVVSANDAGQ
VTSVSQLSDVRPTDWAFQALQSLVERYGCIAGYPDRTYRGNRALTRYEFAAGLNACLDRVNELIAA
STADLVKKEDLATLQTLQEQFAAELATLRGRVDSLEARTTTLEKQQFSTTTKLQGEAIFSISGAQGD
SKAVSSLLERRAAETGAAVARPDVDDNTTFSNRVRLSLRTSFTGQDTLLTRIQARNITDFAGAAGT
NMADLSYEGGRTADNSAFVDKLFYRFPIGQGGAVTIDAINGEFYNNVNNFNPLLASDSQGSISRFG
RFNPIYRQGASGSGSGAGVTLNYPLSQAFTVSLGYLADNGNDPTDKNGLFNGSNAALAQLAFKPS
SNIDLGFTYVRSYDRLTGASVSSGTGSALSNNPFGNFINAGGVRGSAVSADHFGVQGSFKVTPGLV
LSGWGGLTKAHAENRATNADATILNWAVGLAFPDLGGKGNMAGLIFGMPPKVTDSDLVVNGIRQE
DEDTSFHLEGLYRLRLNDKISITPGIIVLFNPEHNNANDTVYVGTIRTTFSF

WP_026148594.1 Carbohydrate-selective porin, OprB family
MKVKKLKFEMQVSLASLVAGSISLAAGNAIAAEPTSELNQIPSVSELSRPTAPTMSQVTSVSQLSDV
RPTDWAFQALQSLVERYGCIAGYPDRTYRGNRALTRYEFAAGLNACLDRVNELIAAATADLVKKED
LATLQKLQEQFAAELATLRGRVDSLEARTSTLEKQQFSTTTKLSGEAIFSVANGYESGDAGNDANT
VFNNRVRLNLNTSFTGKDLLITGLQAQNFGASGIAGGSGSSIAQTLGYADPVFGSNSNVRLSYEPQ
FPTVDPSTLTGKGANNSLSLYKLLYIFPVADKVTAFAGTSAEVSDAFPSVLPWASEGQGALSRFASL
PAAQRVSGGTSQTGLAAAAGVIFNISDAIDFRALYGSVNANIPGNRGLGGGTPLGAGVGGGSYVAA
AQLTVKPSQAFTVALNYAHSYHQINILGTGLSSSDIGAVNLPGGLTLGNLNQGIKMNTLGATAAFRLS
QNITLAGSYSHIFADLVDVDAGTNFNSWLVGLYVTDILKKGNSAGLIFGQPLKRVSTDGIASNPENRT
PYQVEGFFNFRVNDNLSITPGVFAIFNPEGQSTNGTAIVPVIRTTFTF

Fraction1 a

WP_017290631.1 FG-GAP repeat protein
MPGQSILTESFANADVSQLNWLFGTSRDGALPPILTARSNASASAGGLPGGGTDTPGNGVLRLTN
NTQNQGSFVIYDQPINSASGLSVQFDLYAYGGNGADGISFFLIDGAANPTRGGAVGGSLGYSSDST
QSPTVAGLEGGYLGIGFDEFGNFSNTGYGAGGTGFSSQTIAVRGKESLNYQFLTNAKLPGGNRID
VNTSVRSTARRRVGIDLNPNGNLSVSFDVNGNGTLEVDEKLISDFNITQSDRNGALPSTFKFGFAA
STGNQFNFHEVNNLAINTFDGPYIPLVDFDGGTRVIKPSGSFNITATLDVASTQTVTIPLLISGDAIQGI
DYQLSNNFITIAAGQTTGSVTLTGLSNSPTVLDKNLQINLNPPVNAALSPQNLPLNVKLTRMSDNDC
ALPDFNGDNSVDLAWQNSNTNQTAIWLLNGTQVTEAAFTAITGANWRIVSTRDFDADGKTDLLWR
NTSTGENAIWLMDGTSIESTSLIPTLASQNWQVAASRDFNNDGTADIFWVNSATGETEVWLMDGFN
RISATSLITVEAGWQVADVADFNYDGKADLVWRNTRTGENAIWLMNGNAIASAGFITLIPNQDWRIV
AARDTSGDGKADLIWRNDVTGENAIWLMNGITPTSQQFILQTTTPGWRIADVCDTSGDGKADLVW
RNIQTGENAIWLLNGTSLADAGFITQIANQAWAVNGARDTSGDGKADLIWRNNITGETAIWLMDGK
TPTSQQFLFTTEPLDWRAQIRPNAIAL

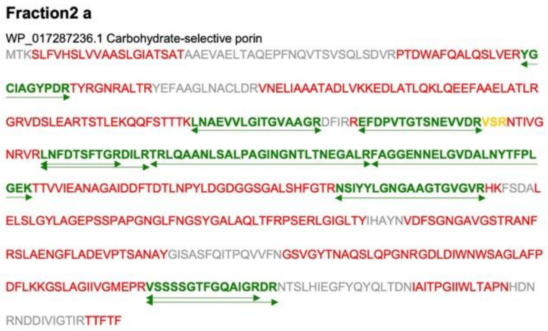

Figure S2. Identification of proteins in Fractions 0, 1, and 2 by mass spectrometry. Amino acid sequences with supported by >95%, 50–95%, and <50% confidence are shown in green, yellow, and red, respectively. Peptide fragments identified with >95% confidence are indicated by horizontal green arrows.

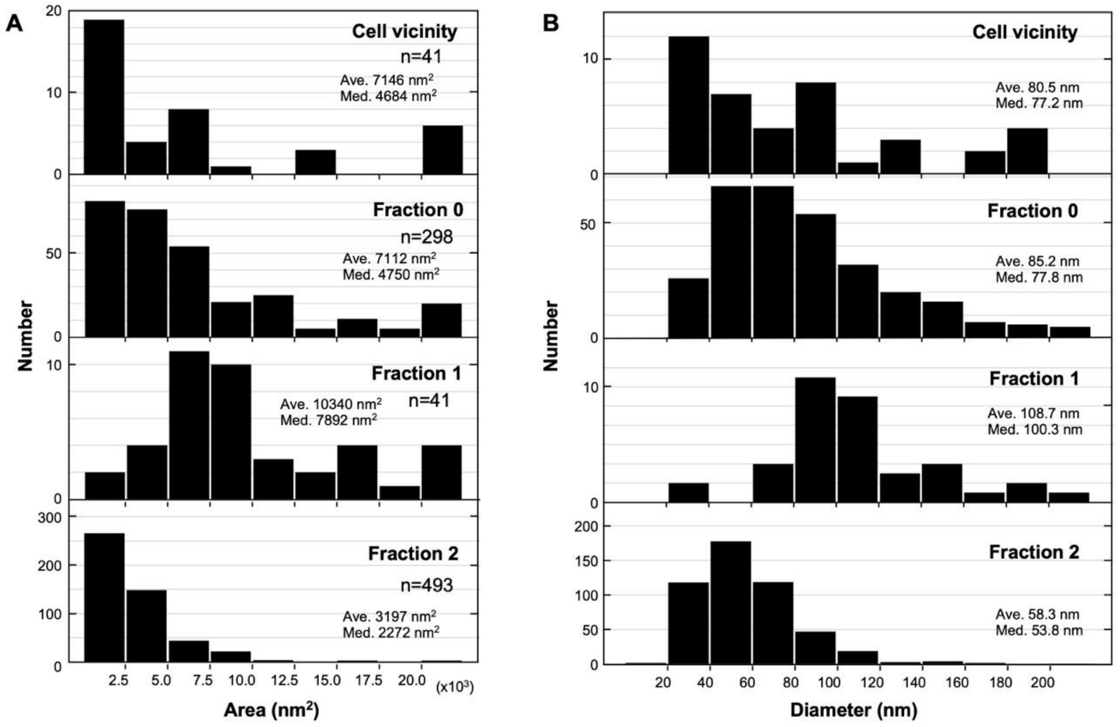

Figure S3. Size distribution of EVs observed in the vicinity of cells and in the Fractions 0, 1, and 2. Panel A shows a histogram of the area of each EV structure; since EVs are not circles, the diameter of the circle corresponding to their area is calculated and its histogram is shown in panel B. 'n' indicates the number of EVs measured (A). Average (Ave.) and median (Med.) values are also shown.

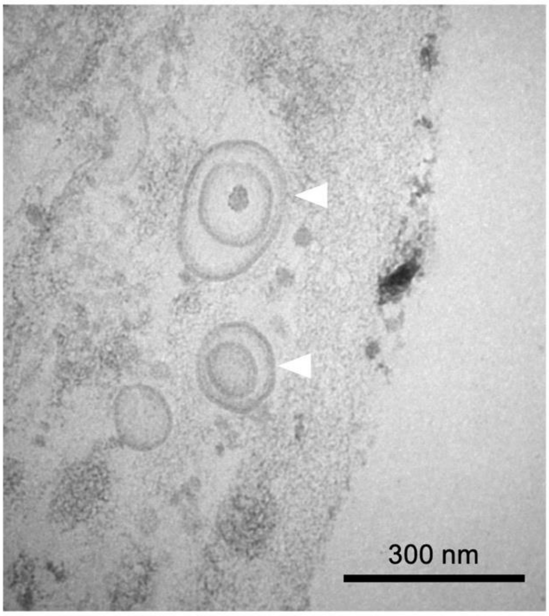

Figure S4. TEM observation of Fraction 1. Multi-lamellar vesicles are shown by white arrow heads.

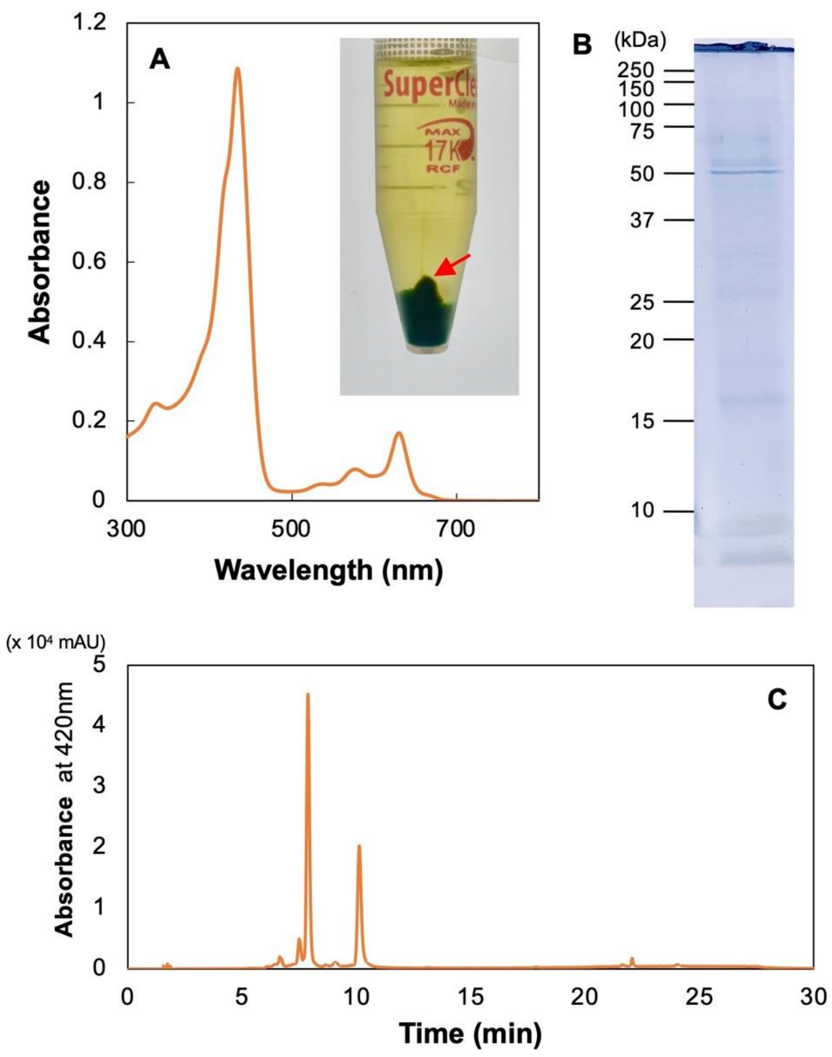

Figure S5. Analysis of dark-green pellets. YFC2 cells grown in the dark were collected by low-speed centrifugation (A, inset). A dark-green pellet (red arrow) was apparently different from cell precipitate. To analyze this dark-green pellet, a portion of the pellet was carefully collected using a small spatula. The collected pellet was suspended in PBS buffer, and then SDS-PAGE (B) and HPLC (C) analyses were performed according to the Materials and Method.
